# Millennials’ Entrepreneurial Values, Entrepreneurial Symbiosis Network and New Ventures Growth: Evidence From China

**DOI:** 10.3389/fpsyg.2021.713280

**Published:** 2021-12-06

**Authors:** Ling Zhang, Xue Zhou, Ekaterina Shirshitskaia

**Affiliations:** Business School, Qingdao University, Qingdao, China

**Keywords:** millennials’ entrepreneurial values, entrepreneurial symbiosis network strength, entrepreneurial symbiosis network scale, new ventures growth, self-verification theory

## Abstract

The fate of new ventures incubated by the same corporate ecosystem is different. Can entrepreneurs’ ideas affect the way out of incubating companies? Based on self-verification theory and symbiosis theory, we took millennial entrepreneurs as the research object, combined with entrepreneurial enterprises’ data in the makerspace. We analyzed the impact of millennials’ entrepreneurial values on new ventures growth and explored the mediating role of entrepreneurial symbiosis networks. The following conclusions are obtained by analyzing the questionnaire of 191 millennial entrepreneurs: Millennials’ entrepreneurial values significantly promote new ventures growth. The strength and scale of the entrepreneurial symbiosis network have a positive impact on new ventures growth. The entrepreneur symbiosis network acts as an intermediary between the millennials’ entrepreneurial values and new ventures growth.

## Introduction

Companies incubated in entrepreneurial ecosystem showed extraordinary results. Is this the ecological result of natural selection? Entrepreneurial ecosystems are different from those of natural ecosystems. Species in the entrepreneurial ecosystem are solitary, and they are entrepreneurs and employees with subjective initiative. Therefore, entrepreneurs will make subjective judgments and strategic adjustments based on changes in the ecological situation, rather than passively adapt and wait for the “survival of the fittest” entrepreneur. Entrepreneurs have absolute control rights within startups, and the entrepreneur’s energy has an extension, which is related to the evolution trend of startups in the makerspace. Entrepreneurs’ ideologies are the basis for their thinking and judgment. We seek to ask the first question: what kind of ideology is conducive to the survival and continuation of entrepreneurial enterprises in the ecosystem? The “post-80s” and “post-90s” millennials have become the backbone of economic development and national construction in China. The 2018 China Startups Growth Index shows that 26–35 years old entrepreneurs constitute 50.42% of Chinese entrepreneurs. Hence, millennials have become a major force for Chinese entrepreneurship. To help develop entrepreneurial enterprises, the government has vigorously created sound entrepreneurial ecology (Tang et al., 2012). Therefore, we seek to ask the second question: what exactly will occur if the millennial entrepreneurs’ ideology collides with the makerspace represented by the makerspace?

Millennial entrepreneurs include the double label of millennials and entrepreneurs. The first is the millennials. This is called the millennials because of its unique imprint. The economic, technological, social, and other aspects of the millennials’ growth period are all pioneering and unique. Economic globalization, internet penetration, and being only children are typical characteristics of millennials. The exceptional growth environment has formed an imprint on the millennial groups. Hence, as a result, they have become the object of public opinion and inquiry. Simultaneously, millennials continue to see opportunities and create achievements in the workplace. Their many advantages have also attracted attention, such as hard work, longing for freedom, and willingness to innovate (Myers and Sadaghiani, 2010). Based on research on millennial workers, some scholars have refined millennial entrepreneurs’ ideology into millennials’ work values, including useful, practical intrinsic preference, interpersonal harmony, innovation orientation, and long-term development (Lyons et al., 2010; Eva et al., 2017). Research has found that millennials’ work values are conducive to improving work performance (Lyons et al., 2010; Garcia et al., 2019). The second label of millennial entrepreneurs is entrepreneurs. Entrepreneurs are a special business group, and are founders and helms of entrepreneurial enterprises related to the life and death choices of new ventures (Carolis et al., 2009).

For this reason, we cannot help but ask: when the millennials are in the position of entrepreneurs, do their work values remain conducive to improving work performance? How can we improve and what is the best course of action? As existing research has not provided an answer (Liu et al., 2019), our study attempts to address this question in the context of makerspace. For the convenience of discussion, this study integrates millennial work values and entrepreneurs as research objects. Moreover, it adjusts the expression method to the millennials’ entrepreneurial values without changing the millennials’ core views.

Work values are individual perceptions of principles, ethics, and beliefs about work, an internal system of thought directly influencing behavior, and the satisfactory output wherein employees expect to achieve at work. The millennials’ work values refer to perceptions of workers born after the 1980s and the 1990s about the principles, ethics, and beliefs of work, and are the work-related criteria used by new generation employees to identify right and wrong and determine preferences (Dose, 1997). Millennials’ entrepreneurial values are an expression of millennials’ work values, which summarize millennial entrepreneurs’ ideology and cognitive elements. Hence, they must reflect value through behavior practice (Hanks, 1993). Therefore, referring to research on emotional cognition or other constructions in the entrepreneurial field (such as entrepreneurial passion, entrepreneurial orientation, and entrepreneurship education), we explore how millennial work values influence new ventures growth using the “attitude-behavior-performance” paradigm (Ho et al., 2011; Shan et al., 2016; Mueller et al., 2017; Jiatong et al., 2021). Considering the platform role of makerspace in the “double entrepreneurship” strategy (a major strategy proposed by China for optimizing innovation), combined with the theoretical discussion of makerspaces in recent studies, we selected the entrepreneurship ecosystem represented by makerspace as the context. Based on both self-verification and symbiosis theories, we explore the mechanism of millennials’ entrepreneurial values, supporting the growth of new ventures. The entrepreneurial symbiosis network in the makerspace clarifies its realization path (Chen et al., 2016; Fu and Li, 2016).

## Theoretical Review and Research Hypotheses

### Entrepreneurship Ecosystem

The makerspace gathers different entrepreneurial activities in a makerspace in a specific geographic area (Autio et al., 2018). Similar to the natural ecosystem, the entrepreneurial ecosystem is the interaction between the community and its habitat. Different life forms constitute other communities, such as the entrepreneur and employee communities at the individual level. Community habitat is a non-biological space wherein various communities depend, including tangible and intangible assets. Entrepreneurial ecosystems, such as makerspace, include infrastructure, capital, social networks, and so on, providing a place for gatherings, experiments, creative transformation, and socializing for groups with common interests. Communities learn and cooperate interactively, share resources, and use a burst of creative sparks in innovation practice (Lakind et al., 2019). Based on the symbiosis theory, they regard the entrepreneurial ecosystem as the totality of community symbiosis and the entrepreneurial symbiosis network as the habitat of community dependence. The growth of new ventures and evolution of the entrepreneurial ecosystem are realized through the interaction between the entrepreneurial community and the entrepreneurial symbiosis network. This process is rooted in symbiotic matrix exchange and symbiotic energy accumulation promoted by different symbiotic units (Boons et al., 2014). It conforms to the needs of innovation and entrepreneurship in the new era and has the characteristics of similar interests, teamwork, and so on (Curley, 2016). Therefore, entrepreneurs construct an ecosystem suitable for growing enterprises and then providing feedback to entrepreneurs through a symbiotic network with entrepreneurs. Moreover, interaction between entrepreneurs and entrepreneurial symbiosis networks is mainly reflected in the influence of entrepreneurial activities based on the subjective consciousness of entrepreneurial symbiosis network characteristics.

### The Millennials’ Entrepreneurial Values and the Growth of New Ventures

Owing to the typical ideology of millennial entrepreneurs, millennials’ entrepreneurial values play a role in growth of new ventures. The essence of their utility is the explicit expression of internal concepts, which echoes the relevant views of self-verification theory. Self-validation theory proposes that individuals pursue behavioral results consistent with their self-concept to maintain or strengthen their self-concept (Swann et al., 1992; Yidong and Xinxin, 2013). Specifically, self-verification includes two stages: the first is constructing behavioral logic based on self-concept and to clarify behavior goals, and the second is strengthening self-concept based on behavior results (Moon et al., 2019). Because of the contextualized nature of entrepreneurial values, millennial entrepreneurs born in the late reform and opening-up period and influenced by the global economy and the Internet era presented unique and multidimensional entrepreneurial values. As per millennials’ unique value cognition, millennials’ entrepreneurial values endow them with a personal self-concept. Millennials’ entrepreneurial values are reflected in practical orientation, pursuing high returns and efficient work and natural preference and are more inclined to choose the position they like, interpersonal harmony and committed to maintaining a harmonious working atmosphere. They are innovation-oriented, more creative, and imaginative, and focused on the long-term development and sustainable development of themselves and the company (Frishammar and Hörte, 2007).

Based on self-verification theory, the entrepreneurial values of millennials help them in the discovery, appropriation, or development of business opportunities to realize the organization’s value. The entrepreneurs actively guide the organization’s members to establish a team atmosphere of cooperation and mutual help. Assistance is conducive to resource coordination and distribution to achieve organizational goals and sustainable development (Tang et al., 2017). Millennial entrepreneurs are pragmatic, innovation-oriented, and focus on long-term development. This means that entrepreneurs with new ideas not only value material rewards and profit maximization but also actively promote generation and implementation of ideas. Moreover, they strive to do their jobs well to build the competitive advantage of the company and achieve long-term development of the venture. Millennials’ entrepreneurial values serve as an internal cognition. Individuals exhibit behaviors consistent with cognition in the workplace (Ping et al., 2011). Therefore, we believe that the values of natural preference and emphasizing efficient work will motivate millennial entrepreneurs to maintain positive self-emotion, which will lead to work behaviors consistent with this self-emotion. This establishes a positive cognitive foundation for millennial entrepreneurs to actively promote entrepreneurial practices, explore the joy of the entrepreneurial process, focus resources on their core business, and drive the growth of new ventures. Accordingly, we hypothesize the following:

**Hypothesize 1 (H1):** The millennials’ entrepreneurial values positively influence the growth of new ventures.

### Millennials’ Entrepreneurial Values and the Entrepreneurial Symbiosis Network

The relationship between millennials’ entrepreneurial values and the entrepreneurial symbiosis network embodies the ecological characteristics of the entrepreneurial ecosystem. This reflects the community’s interaction and community habitat. Among them, millennials’ entrepreneurial values come from the entrepreneurial community, and the entrepreneurial symbiosis network is a community habitat for the survival of the entrepreneurial community. Like the ecosystem concept, symbiosis also originates from biology, which refers to the close connection between different subjects sharing the same breath and fate (Boons et al., 2014). As an ecosystem carrying entrepreneurial companies, R&D institutions, intermediary organizations, and relevant government departments, the makerspace is a form of network survival supporting the sharing of resources, risks, and profits accumulated by the interaction of the above entities. Characteristics such as number of community members, correlations, and heterogeneity of relationships constitute the entrepreneurial symbiotic network structure (Burt, 2004). In this construct, network size, network strength, network scale, centrality, and structural holes are some of the most important variables (Baum and Smith, 2001).

The entrepreneurial symbiosis network can be characterized by two aspects: network strength and network scale. We refer to the related viewpoints of symbiosis theory to define entrepreneurial symbiosis networks’ power and scale (Zeng et al., 2019). The strength of entrepreneurial symbiosis networks refers to the depth of mutual embedding in different makerspaces and subject networks in the community. A high-strength entrepreneurial symbiosis network refers to in-depth interaction between entrepreneurs, employees, and entrepreneurial companies. For example, realizing the organization’s core technology by cooperating with the R&D center in the crowd creation space is necessary. The entrepreneur symbiosis network scale refers to the breadth of the embedded symbiosis interface, which is reflected in the community diversity, resource heterogeneity, and interaction diversity of the entrepreneur symbiosis network. It determines the number of symbiosis units. The larger the network, the more entities that startups can connect with. A large-scale entrepreneurial symbiosis network means that entrepreneurs or entrepreneurial companies receive a high degree of support from channels. For example, entrepreneurs’ financing channels using symbiosis networks include government support funds, banks, venture capital companies, and so on.

Self-verification theory provides that entrepreneurs take actions consistent with their values to gain self-identification (Aryee et al., 2014). Entrepreneurs with millennial entrepreneurial value can effectively support the entrepreneurial symbiosis network. This is mainly reflected in the support for the strength and scale of the entrepreneurial symbiosis network. Regarding the supporting role of millennials’ entrepreneurial values on the power of entrepreneurial symbiosis networks (Twenge et al., 2010), the purpose of symbiotic behavior between startups and other entities in the co-creation space is to make progress together. Under the guidance of millennials’ entrepreneurial values, entrepreneurs actively participated in the construction of entrepreneurial symbiosis networks. Moreover, they conducted in-depth exchanges with stakeholders in the crowd-creation space to realize the expected benefits of resource allocation and knowledge learning, confirm its inherent work preference, and reflect the dual pursuit of utilitarianism and interpersonal harmony (Wang et al., 2010). Second, entrepreneurs actively participate in entrepreneurial symbiosis networks, start-ups, construction of government departments, and institutions involved in research and development, investment and financing, and technology intermediaries (Zhou et al., 2021). These subjects establish contacts to unblock channels to obtain information on the Internet and keep abreast of the dynamic changes of the organization’s internal and external environments to support the construction of their innovative ideas. In this process, each subject uses collective wisdom to solve the problems encountered in development, and the solution of the problem may mean that each subject can obtain external knowledge, and at the same time, it can strengthen its own internal knowledge accumulation to respond to their innovative knowledge needs and long-term development expectations (Audretsch and Belitski, 2017). Therefore, we hypothesize the following:

**Hypothesize 2a (H2a):** The millennials’ entrepreneurial values positively affect the strength of the entrepreneurial symbiosis network.

Based on the theoretical framework of self-verification, entrepreneurs with a new generation of entrepreneurial values will prioritize establishing a harmonious atmosphere of internal and external relations and the long-term benefits of enterprises and industries. Therefore, under guidance of such values, entrepreneurs actively expand the scale of entrepreneurial symbiosis networks. Specifically, entrepreneurs are committed to promoting interpersonal interaction based on the need for interpersonal harmony and guiding entrepreneurs to connect with as many stakeholder groups as possible, such as governments, banks, and other enterprises, to expand access to information and resources and build a broad and stable interactive symbiosis network (Shao et al., 2011). Second, based on long-term development needs, entrepreneurs strengthen their insights into stakeholders in the makerspace. Based on keen entrepreneurial vigilance, we can influence entrepreneurs to interpret policy information and extra-organizational behaviors. Combine entrepreneurs’ development power to stimulate impromptu relationship-building actions and achieve dynamic adjustment and expansion of the symbiosis network (Strobl and Kronenberg, 2016). Third, based on innovation-oriented needs, entrepreneurs are committed to expanding the scale of the entrepreneurial ecosystem. The larger the scale of the co-creation space, the richer the symbiosis matrix (Huang et al., 2017). The rich symbiosis matrix means that there are a lot of resources and information in the co-creation space, which is conducive to start-up companies to make up for their lack of resources, and to gain an advantage for corporate innovation. Accordingly, we hypothesize the following:

**Hypothesize 2b (H2b):** The millennials’ entrepreneurial values positively affect the scale of entrepreneurial symbiosis networks.

In summary, we hypotheses:

**Hypothesize 2 (H2):** The millennials’ entrepreneurial values positively affect the characteristics of the entrepreneurial symbiosis network.

### The Mediating Effect of Entrepreneurial Symbiosis Networks

Each subject in the symbiosis network establishes a coexistence relationship. This aims to produce a “1 + 1 > 2” symbiosis effect and promote expected growth in both (Bosch-Sijtsema and Bosch, 2015). From the perspective of the strength of entrepreneurial symbiosis networks, its supporting role in developing new ventures is analyzed. On the one hand, strong symbiotic relationships are conducive to the generation of core symbiosis energy. Subjects with strong symbiotic relationships had mutual recognition and in-depth cooperation. Interaction occurs on the organization’s core resources, such as technology, to generate symbiotic points such as new technologies provide the impetus for new startup growth. For example, companies in the same makerspace conduct joint research and development to achieve economies of scale or scope. This reduces operating costs and risks. Especially under the new wave of technological revolution, enterprises through online and offline communication and interaction jointly innovate, research and develop, produce products or provide services, raise funds, and incubate self-organized innovation and entrepreneurship activities. Hence, they form a mass innovation space (Batjargal et al., 2013). On the other hand, a robust symbiotic relationship improves efficiency of symbiotic behavior and accelerates new startup growth. Exchanging information and resources in the symbiotic network is interactive in two ways. The strong symbiosis relationship enables startups to obtain real and practical essential information when transmitting data and feedback to other subjects in the co-creation space. Therefore, entrepreneurial enterprises can identify and seize opportunities to realize emerging entrepreneurs’ growth (Chen et al., 2016). Accordingly, we hypothesize the following:

**Hypothesis 3a (H3a):** The strength of the entrepreneurial symbiosis network plays an intermediary role in millennials’ entrepreneurial values and new ventures growth.

From the perspective of the scale of the entrepreneurial symbiosis network, we analyze its supporting role in the growth of new ventures. A large-scale entrepreneurial symbiosis network is beneficial to entrepreneurial enterprise development. This provides a rich symbiotic substrate for forming symbiotic attraction and stimulating entrepreneurial enterprises to adopt symbiotic behaviors to support their development (Chen et al., 2016). The more varied the subjects in the makerspace symbiosis network are, the more resources new ventures can incorporate from the makerspace. Start-up companies integrate resources based on corporate development logic or use corporate influence to obtain business development opportunities (Xu et al., 2019). Second, the massive scale of the entrepreneurial symbiosis network helps form a multidimensional symbiosis interface architecture. The entrepreneur’s symbiosis network includes various heterogeneous symbiosis interfaces, including technology research and development, talent training, capital flow, and knowledge interaction. By incorporating unique resources, such as technology trading centers in makerspaces or mutual guarantee agencies, into the symbiosis interface, capital accumulation, technological innovation, or knowledge update can be achieved and entrepreneurial behavior and enterprise development can be promoted (Gawer, 2020). Accordingly, we hypothesize the following:

**Hypothesize 3b (H3b):** The scale of entrepreneurial symbiosis network plays an intermediary role in the millennials’ entrepreneurial values and the growth of new ventures.

In summary, we hypotheses:

**Hypothesize 3 (H3):** The entrepreneurial symbiosis network characteristics play an intermediary role in the millennials’ entrepreneurial values and the growth of new ventures.

**FIGURE 1:**
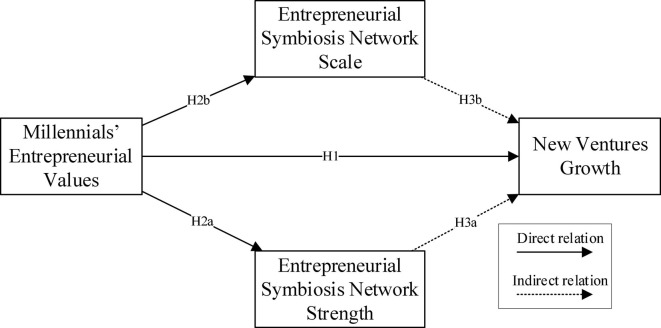
Conceptual model.

The theoretical model of this article is shown in Figure 1.

## Researrch Design

### Sample Selection and Data Collection

This study used field surveys to collect the questionnaires. Data was collected from May to September 2019. Samples mainly came from the crowd-creation spaces in the eastern entrepreneurial region of China, which enjoyed relatively high financial support, with the advantages of a sufficient supply of raw materials and many engineering universities nearby. Before implementing the survey, a pilot test was applied by distributing 75 questionnaires to the new ventures in Shandong province in order to identify any problems regarding the wording, content and ambiguity of the questions. The pilot test lasted from March to April 2019. As a result, 61 questionnaires were received with a participation rate of 81%. The reliability and validity of the measurement constructs were acceptable.

According to Zahra’s (1993) research, this study considered companies with fewer than 8 years as entrepreneurial companies. The specific method of questionnaire collection was as follows: after researchers contacted the responsible agency of makerspace, one researcher collected data on the spot by visiting companies and issuing the questionnaires, and the other requested makerspace staff to share the link to the questionnaire in the online group of makerspace entrepreneurs and collected the results online. We used three methods to minimize general method deviation. The first was by implementing anonymous filling methods to reduce the responsibilities of the personnel filling the questionnaire. The second was to avoid the questionnaire being filled out multiple times by the same person (filling in online and offline questionnaires at once). The third required the same startup company to collect only one sample to avoid data from comparing the same company subjects. Ultimately, this survey returned a total of 247 questionnaires and excluded incomplete or overlapping questionnaires. Only questionnaires of millennial entrepreneurs born in the 1980s and the 1990s were retained. 191 valid questionnaires were obtained, with a recovery rate of 77.33%.

Among the test participants, 34.3% were women. The proportion of men was 65.7%, and the educational background distribution was 9.5% for college and below, 70.6% for undergraduates, 17.4% for master’s degree students, and 2.5% for doctoral students. As the research object involves includes entrepreneurs, the subjects’ ages were mainly distributed between 20 and 30 years old and 30–40 years old, which account for 50.8 and 49.2%, respectively.

### Variable Measurement

For the adopted foreign scales, a translation and back translation process was adopted to avoid semantic deviation. The questionnaire measurement of variables in this study utilizes a seven-point Likert scale with 1 indicating complete disagreement and 7 indicating complete compliance. The necessary information of the enterprise is measured using the form of selection or filling in the blanks.

#### The Millennials’ Entrepreneurial Values

Drawing on research results of Lyons et al. (2010), 20 items were used for measuring millennials’ entrepreneurial values, including practical orientation, intrinsic preference, interpersonal harmony, innovation orientation, and long-term development. The internal consistency of the Cronbach’s α coefficient was 0.861.

Entrepreneurship symbiosis network strengths Learning from Capaldo (2007), four items were used to measure entrepreneurship symbiosis network strength. These include interaction frequency, resource input, cooperation content, and reciprocity. The internal consistency of the Cronbach’s α coefficient was 0.735.

#### Entrepreneurship Symbiosis Network Scale

Drawing on the research of Eisingerich et al. (2010), three items were used to measure the entrepreneurship symbiosis network scale. One example would be “the amount of support from banks and other investment institutions obtained through makerspace.” The internal consistency of the Cronbach’s α coefficient was 0.664.

#### Growth of New Ventures

Drawing on the research of Hmieleski and Baron (2008) and Stam and Elfring (2008) and the measurement indicators of growth performance (Hanks, 1993), the following five items are used to measure the growth of new ventures: financial growth, employee increase, customer satisfaction improvement, corporate reputation growth, and new product or service growth. The internal consistency of the variable Cronbach’s α coefficient is 0.787.

#### Control Variables

According to previous studies, the personal characteristics of entrepreneurs and that of startups affect the growth of new entrepreneurial enterprises. Concerning existing research practices, the entrepreneur’s gender, age, educational background, previous experience, and firm age and size are control variables.

## Empirical Analysis and Results

### Convergent Validity

Convergent validity was a measure of the model fit. The average variance extracted (AVE) showed the degree of correlation between the construct and its indices, with a greater fit achieved with stronger correlation. Any composite-reliability (CR) rating higher than 0.7 (Chin, 1998) suggests that the construct was internally acceptable. In this study, the AVE of all variables was higher than 0.5 and the CR of all variables was higher than 0.7 (Table 1).

**TABLE 1:** Indicators of measurement.

**Variable**	**Items**	**Factor loading**	**Average variance extracted (AVE)**	**Composite reliability (CR)**	**Cranach’s alpha**
Millennials’ entrepreneurial values	EV1	0.631	0.517	0.954	0.861
	EV2	0.683			
	EV3	0.580			
	EV4	0.497			
	EV5	0.508			
	EV6	0.563			
	EV7	0.924			
	EV8	0.703			
	EV9	0.621			
	EV10	0.692			
	EV11	0.627			
	EV12	0.588			
	EV13	0.950			
	EV14	0.583			
	EV15	0.814			
	EV16	0.844			
	EV17	0.950			
	EV18	0.762			
	EV19	0.806			
	EV20	0.785			
Entrepreneurial symbiosis network strength	NS1	0.775	0.561	0.836	0.735
	NS2	0.730			
	NS3	0.776			
	NS4	0.712			
Entrepreneurial symbiosis network scale	NH1	0.741	0.570	0.799	0.664
	NH2	0.792			
	NH3	0.730			
Growth of new ventures	NG1	0.583	0.538	0.851	0.787
	NG2	0.671			
	NG3	0.852			
	NG4	0.679			
	NG5	0.845			

### Discriminant Validity

Using AMOS22.0 to test discriminant validity of the variables involved, we conducted confirmatory factor analysis of the millennials’ entrepreneurial values, strength of entrepreneurial symbiosis networks, scale of entrepreneurial symbiosis networks, and growth of new ventures. The results of the AMOS confirmatory factor analysis are then presented in Table 2. The data fit of the four-factor model (χ^2^/df = 1.54, RMSEA = 0.05, SRMR = 0.05, CFI = 0.94, TLI = 0.93) was the most ideal, which was significantly better than that of the other models. Results showed that the four variables involved in this study had good discriminant validity.

**TABLE 2:** Confirmatory factor analysis results.

**Models**	**χ^2^**	**df**	**χ^2^/df**	**RMSEA**	**SRMR**	**CFI**	**TLI**
Four factors	129.43	84	1.54	0.05	0.05	0.94	0.93
Three factors^a^	149.61	87	1.72	0.06	0.06	0.92	0.90
Two factors^b^	207.11	89	2.33	0.08	0.07	0.85	0.82
One factor^c^	246.21	90	2.74	0.10	0.08	0.80	0.77

*^*a*^Millennials*’ *Entrepreneurial Values + Entrepreneurial Symbiosis Network Strength, Entrepreneurial Symbiosis Network Scale, New Ventures growth.*

*^*b*^Millennials*’ *Entrepreneurial Values + Entrepreneurship Symbiosis Network Strength + Entrepreneurial Symbiosis Network Scale, New Entrepreneurial Growth.*

*^*c*^Millennials*’ *Entrepreneurial Values + Entrepreneurial Symbiosis Network Strength + Entrepreneurship Symbiosis Network Scale + New Ventures Growth.*

### Descriptive Statistical Analysis

Descriptive statistics mainly display average value, standard deviation, and correlation coefficient of each variable (Table 3). This study uses the entrepreneur’s gender, age, educational background, previous experience, firm age, and firm size as control variables. According to the correlation analysis results, a significant positive correlation was found between millennials’ entrepreneurial values and new ventures growth (*r* = 0.45, *p* < 0.01). A significant positive correlation was found between millennials’ entrepreneurial values and entrepreneurial symbiosis network strength (*r* = 0.56, *p* < 0.01) and the entrepreneurial symbiosis network scale (*r* = 0.37, *p* < 0.01). The results provide specific support for subsequent hypothesis argumentation in this study.

**TABLE 3:** Descriptive statistical analysis.

	**Mean**	***SD***	**1**	**2**	**3**	**4**	**5**	**6**	**7**	**8**	**9**
Gender	1.32	0.47									
Age	2.49	0.50	0.10								
Educational background	4.09	0.72	0.08	0.18*							
Firm age	3.59	1.90	0.01	0.28**	0.05						
Previous experience	5.65	2.87	−0.01	0.58**	0.21**	0.40**					
Firm size	3.54	1.66	0.19**	0.21**	0.16*	0.41**	0.31**				
Millennials’ entrepreneurial values	5.39	0.71	0.05	0.15*	0.08	−0.09	0.14	0.01			
Entrepreneurial symbiosis network strength	5.24	0.97	0.04	0.10	−0.04	0.05	0.18*	0.17*	0.56**		
Entrepreneurial symbiosis network scale	4.83	1.14	0.06	0.06	0.02	0.08	0.09	0.20**	0.37**	0.27**	
New ventures growth	4.61	1.01	0.13	0.07	−0.01	0.07	0.13	0.24**	0.45**	0.56**	0.42**

*Significantly correlated at the 0.05 level (bilateral); **significantly correlated at the 0.01 level (bilateral).

### Common Method Bias

Common method bias often arises when the questionnaire method is used for data collection. This questionnaire adopts an anonymous evaluation method; however, common method bias for the same participants remains unavoidable. To test the common method bias, we used Harman’s single factor test to perform an unrotated factor analysis on all collected questionnaire item data. The variance explained by the first principal component is 27.01%. This does not constitute half of the variance explained by the total variable (67.12%). Therefore, the common method bias of the sample data was within an acceptable range.

### Hypothesis Testing

Using SPSS22.0 software, hypothesis testing was performed after controlling for gender, age, educational background, previous experience, company age, company size, and other related variables. Table 4 present the output results of the statistical analysis.

**TABLE 4:** Regression analysis between variables.

**Outcome variable**	**Predictor variable**	**β**	***t***	***R***	**R-sq**	***F***
NG	Gender	0.077	0.193	0.523	0.273	9.831***
	Educational background	−0.075	−0.443			
	Age	−0.072	−0.403			
	Firm age	0.029	0.665			
	Previous experience	0.038	−0.216			
	Firm size	0.228	2.556***			
	EV	0.458	5.477**			
NS	Gender	0.004	0.058	0.598	0.358	14.554***
	Educational background	−0.115	−1.875			
	Age	−0.071	−0.955			
	Firm age	0.022	0.311			
	Previous experience	0.113	1.436			
	Firm size	0.150	2.208**			
	EV	0.562	9.229***			
NH	Gender	0.013	0.193	0.422	0.178	5.667***
	Educational background	−0.031	−0.443			
	Age	−0.034	−0.403			
	Firm age	0.052	0.665			
	Previous experience	−0.019	−0.216			
	Firm size	0.196	2.556**			
	EV	0.377	5.477***			
NG	Gender	0.076	1.251	0.378	0.351	13.838***
	Educational background	−0.028	−0.461			
	Age	−0.043	−0.593			
	Firm age	0.020	0.298			
	Previous experience	−0.008	−0.101			
	Firm size	0.167	2.469			
	EV	0.231	3.176**			
	NS	0.404	5.542***			
NG	Gender	0.074	1.170	0.323	0.294	10.874***
	Educational background	−0.067	−1.061			
	Age	−0.064	−0.835			
	Firm age	0.016	0.227			
	Previous experience	0.043	0.524			
	Firm size	0.179	2.527			
	EV	0.365	5.399***			
	NH	0.247	3.671***			

N = 191, *** and ** indicate p < 0.001 and p < 0.01, respectively.

*EV, millennials’ entrepreneurial values; NG, growth of new ventures; NS, entrepreneurial symbiosis network strength; NH, entrepreneurial symbiosis network scale.*

**FIGURE 2:**
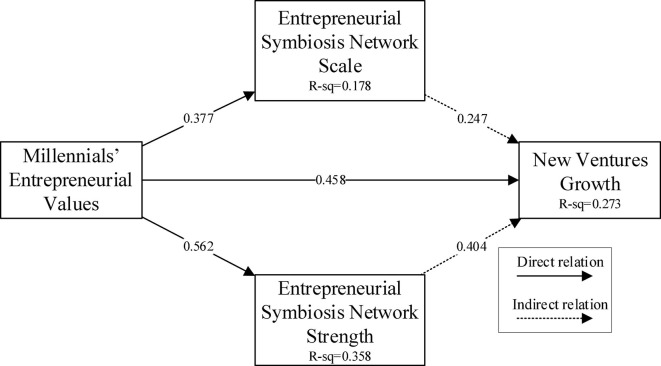
Regression results for hypotheses testing.

In direct effects, the influence coefficient of millennials’ entrepreneurial values on the growth of new ventures (β = 0.458, *t* = 5.477, *p* < 0.01) and on the strength of the entrepreneurial symbiosis network (β = 0.562, *t* = 9.229, *p* < 0.001) and the millennials’ entrepreneurial values on the scale of entrepreneurial symbiosis network (β = 0.377, *t* = 5.477, *p* < 0.001) were all significantly positive. Therefore, H1, H2, H2a, and H2b were supported. Specifically, when entrepreneurs have millennials’ entrepreneurial values, the growth of new ventures is better. Second, when entrepreneurs have millennials’ entrepreneurial values, the greater the strength and scale of the entrepreneurial symbiosis network. From the perspective of the mediation effect, the influence coefficient of millennials’ entrepreneurial values on the growth of new ventures through the strength of the entrepreneurial symbiosis network (β = 0.231, *t* = 3.176, *p* < 0.01) was significantly positive. The influence coefficient of millennials’ entrepreneurial values on the growth of new ventures through the scale of entrepreneurial symbiosis network (β = 0.365, *t* = 5.399, *p* < 0.001) was significantly positive. Therefore, the strength of the entrepreneurial symbiosis network and the entrepreneurial symbiosis network’s scale play an intermediary role between millennials’ entrepreneurial values and the growth of new ventures. H3, H3a, and H3b were supported. Figure 2 presents the results of regression analysis for better understanding.

## Conclusion and Discussion

### Main Research Conclusion

From a basic biological perspective, the interaction between the population and its living environment forms an ecosystem. Hence, attention is paid to the typical entrepreneurial ecosystem makerspace, the new generation of entrepreneurial values is analyzed, entrepreneurs are encouraged to establish a symbiosis network, and new ventures are launched. This follows the theoretical logic of “attitude-behavior-performance.” Based on both self-verification and symbiosis theories, taking millennial entrepreneurs in makerspace as the research object, we can draw the following three conclusions.

First, entrepreneurs with millennials’ entrepreneurial values are conducive to the growth of new businesses. This conclusion shows that millennial entrepreneurs are dominant in Chinese entrepreneurship. Their unique millennial entrepreneurial values are conducive in supporting new enterprises’ development. This echoes the conclusion highlighted by Garcia et al. (2019) in their research that millennial entrepreneurial values improve job performance. To a certain extent, this explains the different versions of startups incubated in the same makerspace. Millennials who grew up in the context of rapid economic development and reform and opening-up have established the concept of “life is toss.” This is successfully embodied in entrepreneurial activities with the logic of self-verification, which is quite suitable for uncertain characteristics of entrepreneurial activities. Guided by millennial entrepreneurs’ values, millennial entrepreneurs have greater aspirations, and we hope to continuously identify and respond to risks in the vague and complex business environment and promote new ventures growth.

Second, entrepreneurs with millennials’ entrepreneurial values help strengthen and expand the entrepreneurial symbiosis network. This conclusion shows that the characteristics of entrepreneurial communities may affect the community environment in which they live and clarify that the community characteristics of millennial entrepreneurial values and have positive impact on the entrepreneurial symbiosis network. Basically, millennial entrepreneurship values can guide entrepreneurs in conducting entrepreneurial activities benefiting the entrepreneurial symbiosis network, thereby establishing an ecosystem conducive to the symbiosis of different communities in the makerspace. Under the guidance of millennials’ entrepreneurial values, entrepreneurs actively seek cooperation opportunities for mutual assistance and joint development with other symbiotic units in makerspace to strengthen entrepreneurial enterprises and other stakeholders (de Oliveira and Cortimiglia, 2017). Moreover, entrepreneurs with millennials’ entrepreneurial values can encourage them to expand their symbiosis network, enrich types of symbiosis units in the entrepreneurial symbiosis network, and expand the scale of the entrepreneur symbiosis network.

Third, the entrepreneurial symbiosis network plays an intermediary role between millennials’ entrepreneurial values and new enterprises’ growth. The essence of makerspace is the interaction between entrepreneurs and other communities and their habitats. The relationship between entrepreneurs and the entrepreneurial symbiosis network is a macroscopic manifestation of the above effects. Therefore, under millennials’ entrepreneurial value guidance, entrepreneurs have conducted entrepreneurial activities conducive to the entrepreneurial symbiosis network. They improve the community environment for entrepreneurs and entrepreneurial companies and will inevitably promote new venture capital growth. This conclusion is consistent with those highlighted by previous studies. That is, embedding in the crowd to create a spatial network can promote development of entrepreneurial enterprises (Zhang and Bartol, 2010). On the one hand, by strengthening the corporate symbiosis network and integrating symbiosis energy based on in-depth interaction between corporate symbiosis units, we support new enterprises in completing key links, such as new product research, thereby promoting new venture development. On the other hand, by expanding the entrepreneur symbiosis network’s scale, we enrich the symbiosis matrix in the entrepreneur symbiosis network, establish multiple symbiosis interfaces, expand resource entrepreneurs’ acquisition and information exchange channels, and realize the development of new ventures.

### Theoretical Contribution

First, we interpret the growth differences of new ventures in the makerspace from entrepreneurs’ ideology. Previous studies have mainly focused on theoretical discussions or case studies on the evolution of makerspaces and incubators (Engel et al., 2017). Many summaries and discussions on the development of entrepreneurial activities in the evolution of makerspaces were found. However, most lack support of empirical data. This research is based on the collection of entrepreneurs’ and entrepreneurial enterprise data in the makerspace to reflect entrepreneurial enterprises’ survival status in the makerspace. We combined both self-verification and symbiosis theories to explain the millennials’ entrepreneurial values’ mechanism to help the growth of incubating enterprises. This deepens research on the makerspace and enriches the application context of symbiosis theory.

Second, the interdisciplinary integration of the “attitude-behavior-performance” entrepreneurial research paradigm and biological ecosystem theory was realized. In the past, many studies were based on the “attitude-behavior-performance” paradigm, which focus on the impact of both entrepreneurial passion and orientation on entrepreneurial activities (Batjargal et al., 2013; Tang et al., 2015; Ge et al., 2019). Following this framework, millennials’ entrepreneurial values must act on entrepreneurial performance through entrepreneurial practice. Focusing on the makerspace’s particular circumstances, interaction between millennials’ entrepreneurial values and entrepreneurial symbiosis network adheres to the biological viewpoint wherein the ecosystem is the interaction between the community and the community habitat. This study combines the above two perspectives to discuss the relationship between millennials’ entrepreneurial values, entrepreneurial symbiosis networks, and new ventures growth. This deepens the intersection of entrepreneurial and ecological research.

### Management Enlightenment

First, entrepreneurs should boldly change their ideas and develop millennial’s entrepreneurial values. Entrepreneurs are working hard to develop entrepreneurial activities in a complex and dynamic business environment. The tenacity of entrepreneurial will and correct values are the driving forces for entrepreneurs to move forward. Millennial entrepreneurs who grew up during the reform and opening-up had unique values. Through theoretical analysis and empirical research, millennial entrepreneurs with millennial entrepreneurial value are found to be beneficial to the growth of entrepreneurial enterprises. Therefore, millennial entrepreneurs should be confident in their intergenerational peculiar values, maintain the pioneering spirit of millennials’ unique personalities and daring to take risks and put them into practice.

Second, startups should actively integrate into the makerspace, such as the makerspace, and build a mutually beneficial symbiotic relationship with other entities in the makerspace to achieve sustainable entrepreneurship. Startup companies had new problems. Various companies have built multiple types of makerspaces to support them in implementing the double innovation strategy. This study analyzes entrepreneurial companies in the makerspace and finds that establishing close and extensive symbiotic network relationship with various entities in the makerspace can significantly support new ventures growth. Entrepreneurs should fully understand the characteristics of entrepreneurial companies and those of multiple makers’ spaces and settle in the makers’ areas that match the company’s positioning and development vision. In this way, we leverage the makers’ space ecology and strengthen the resource interaction and information interaction of government agencies, R&D institutions, platform organizations, upstream and downstream enterprises, and so on. In the makerspace, we used multiple symbiotic media to construct symbiotic interfaces, generate symbiotic energy, and seek entrepreneurial enterprises’ development.

### Shortcomings and Prospects

This study has the following limitations, which can be improved in future research.

First, we design theoretical models based on the theoretical framework of the attitude–behavior –performance in speculative model design. From the perspective of millennials’ entrepreneurial values, this study explores the mechanism of its effect on startup development. Future research can reflect entrepreneurship compared to different entrepreneurial attitude constructs (such as entrepreneurial orientation, entrepreneurial passion, and millennials’ entrepreneurial values), influence the mechanism of entrepreneurial practice, clarify the effect of entrepreneurial mindset on entrepreneurial behavior, and guide entrepreneurs to construct entrepreneurial ideas.

Second, in the universality of the research conclusions, the samples used in this study are mainly derived from typical co-maker spaces such as Hangzhou Dream Town and ignore the entrepreneurial entities in the small-scale co-creation spaces. Therefore, whether the research conclusions are suitable for small-scale co-creation spaces still needs further empirical analysis. Moreover, the nature of this study was a cross-sectional design, and it did not consider the impact of the time for startups to move into co-maker spaces on their entrepreneurial values and the maturity of their entrepreneurial symbiosis network. Future research could comprehensively consider the scale and time of entry of new ventures into the co-maker space, and broaden the data source to make a more reasonable design.

Third, considering the scale’s scientific nature, the millennials’ entrepreneurial values scale is derived from millennial employees. Although this reflects the uniqueness of millennials, the characteristics of the entrepreneurs studied are not considered. Future studies can develop a dedicated scale around millennial entrepreneurs to accurately reflect the entrepreneurial attitude of millennial entrepreneurs.

## Data Availability Statement

The raw data supporting the conclusions of this article will be made available by the authors, without undue reservation.

## Author Contributions

XZ and LZ: conceptualization. XZ: methodology, software, resources, writing—original draft preparation, visualization, supervision, project administration, and funding acquisition. LZ: validation, formal analysis, investigation, and data curation. LZ and ES: writing—review and editing. All authors have read and agreed to the published version of the manuscript.

## Conflict of Interest

The authors declare that the research was conducted in the absence of any commercial or financial relationships that could be construed as a potential conflict of interest.

## Publisher’s Note

All claims expressed in this article are solely those of the authors and do not necessarily represent those of their affiliated organizations, or those of the publisher, the editors and the reviewers. Any product that may be evaluated in this article, or claim that may be made by its manufacturer, is not guaranteed or endorsed by the publisher.
